# Zinc accumulation aggravates cerebral ischemia/reperfusion injury by promoting inflammation

**DOI:** 10.3389/fncel.2023.1065873

**Published:** 2023-03-08

**Authors:** Wei Li, Xueqi Yang, Mao Ding, Wenjuan Shi, Yuyou Huang, Qi An, Zhifeng Qi, Yongmei Zhao

**Affiliations:** ^1^Institute of Cerebrovascular Diseases Research, Xuanwu Hospital of Capital Medical University, Beijing, China; ^2^Beijing Geriatric Medical Research Center, Beijing, China

**Keywords:** zinc, inflammation, middle cerebral artery occlusion, cerebral ischemia, apoptosis

## Abstract

Intracellular zinc accumulation has been shown to be associated with neuronal death after cerebral ischemia. However, the mechanism of zinc accumulation leading to neuronal death in ischemia/reperfusion (I/R) is still unclear. Intracellular zinc signals are required for the production of proinflammatory cytokines. The present study investigated whether intracellular accumulated zinc aggravates I/R injury through inflammatory response, and inflammation-mediated neuronal apoptosis. Male Sprague–Dawley rats were treated with vehicle or zinc chelator TPEN 15 mg/kg before a 90-min middle cerebral artery occlusion (MCAO). The expressions of proinflammatory cytokines TNF-α, IL-6, NF-κB p65, and NF-κB inhibitory protein IκB-α, as well as anti-inflammatory cytokine IL-10 were assessed at 6 or 24 h after reperfusion. Our results demonstrated that the expression of TNF-α, IL-6, and NF-κB p65 increased after reperfusion, while the expression of IκB-α and IL-10 decreased, suggesting that cerebral ischemia triggers inflammatory response. Furthermore, TNF-α, NF-κB p65, and IL-10 were all colocalized with the neuron-specific nuclear protein (NeuN), suggesting that the ischemia-induced inflammatory response occurs in neurons. Moreover, TNF-α was also colocalized with the zinc-specific dyes Newport Green (NG), suggesting that intracellular accumulated zinc might be associated with neuronal inflammation following cerebral I/R. Chelating zinc with TPEN reversed the expression of TNF-α, NF-κB p65, IκB-α, IL-6, and IL-10 in ischemic rats. Besides, IL-6-positive cells were colocalized with TUNEL-positive cells in the ischemic penumbra of MCAO rats at 24 h after reperfusion, indicating that zinc accumulation following I/R might induce inflammation and inflammation-associated neuronal apoptosis. Taken together, this study demonstrates that excessive zinc activates inflammation and that the brain injury caused by zinc accumulation is at least partially due to specific neuronal apoptosis induced by inflammation, which may provide an important mechanism of cerebral I/R injury.

## 1. Introduction

Zinc (Zn^2+^) is essential for normal cellular functions and plays a signaling role in the brain (Qi et al., 2016; Mutlu and Baltaci, 2020; Alvarez et al., 2021). Zn^2+^ influx from the extracellular space and its mobilization from intracellular pools such as mitochondria, lysosomes, and cytosolic Zn^2+^ binding proteins lead to neuronal damage (Galasso and Dyck, 2007; Sensi et al., 2011). A growing number of basic and clinical experiments have suggested that cerebral ischemia/reperfusion (I/R) caused cellular injury resulting from activation of Zn^2+^ accumulation (Guo et al., 2019; Zhao et al., 2021; Manzanero et al., 2013). Removing Zn^2+^ with a specific Zn^2+^ chelator N,N,N’,N’-tetrakis (2-pyridylmethyl) ethylenediamine (TPEN) reduced Zn^2+^ accumulation in ischemic neurons, rescued them from cell death and improved functional outcomes (Qi et al., 2019). These findings implicate that Zn^2+^ acts as a critical mediator of neuronal death at high concentrations in the ischemic condition. In recent study, we showed that the overload of Zn^2+^ in mitochondria disrupted the function of mitochondria, resulting in reactive oxygen species generation and triggering cell death (Zhao et al., 2018; Qi et al., 2019). Reducing Zn^2+^ accumulation in mitochondria contributes to decreased cerebral ischemic injury by normobaric hyperoxia treatment (Dong et al., 2015). We also found that intracellular accumulated Zn^2+^ aggravates I/R injury through inducing endoplasmic reticulum stress (Zhao et al., 2022). However, the reason why Zn^2+^ produces severe brain damage remains to be elucidated.

Inflammation and immune responses have been proven to be crucial factors associated with stroke onset and progression (Lively et al., 2016; Li T. et al., 2022). In recent years, several cytokines have been proven especially promising as potential therapeutic targets for experimental ischemic stroke including the tumor necrosis factor-α (TNF-α), interleukin-6 (IL-6), and the anti-inflammatory cytokine-10 (IL-10; Zhang et al., 2017; Lambertsen et al., 2019). TNF-α is a proinflammatory factor and works as an initiation factor of inflammatory response. The expression of TNF-α is rapidly upregulated after cerebral ischemia, leading to neurotoxicity (Vila et al., 2000; Shen et al., 2022). As a nuclear transcription factor, nuclear factor kappa B (NF-κB) constitutes the most basic NF-κB signaling pathway with IκB and IκB kinase complex, which is involved in the regulation of inflammation (Chen et al., 1998; Berti et al., 2002). In I/R injury, NF-κB is activated by a variety of stimuli including TNF-α (Jarosz et al., 2017; Kang et al., 2019). Production of TNF-α and activation of NF-κB have been documented to play critical roles in the process of cerebral disease (Hussain et al., 2016). Besides, the activation of NF-κB is also regulated by IL-6 (Jarosz et al., 2017; Kang et al., 2019). IL-6 is a pleiotropic cytokine involved in many central nervous system disorders including stroke. The expression of IL-6 is most prominently identified in neurons in the peri-ischemic regions (Kumari et al., 2016; Rasmussen et al., 2019; Ridwan et al., 2021). In contrast to IL-6, IL-10 is a pleiotropic anti-inflammatory cytokine, which binds to IL-10 receptors to reduce inflammation and limit apoptosis (Chen et al., 2013). Therapeutic administration of IL-10 has been shown to be neuroprotective in experimental stroke and to limit post-stroke inflammation (Chen et al., 2013; Garcia et al., 2017).

The immune system, especially the inflammation, is markedly susceptible to changes of Zn^2+^ levels (Haase and Rink, 2014). Zn^2+^ induces the synthesis of DNA, RNA, and proteins to meet the desired immune response (Baltaci et al., 2019). Recent reports have indicated that Zn^2+^ homeostasis affects neuroinflammation in the brain (Baltaci et al., 2022). Intracellular Zn^2+^ signals are required for the production of proinflammatory cytokines IL-6 and TNF-α, which are also directly induced by incubation with high extracellular Zn^2+^ concentrations (Haase and Rink, 2014; Olechnowicz et al., 2018). Also, in type 2 diabetes mellitus patients, impaired Zn^2+^ homeostasis leads to uncontrolled expression of immune mediators, such as IL-6 and NF-κB, which simultaneously exacerbate the immune response (Bonaventura et al., 2015; Olechnowicz et al., 2018). Zn^2+^ is necessary for the activation of NF-κB signaling pathway, whereas chelating Zn^2+^ with membrane permeable Zn^2+^ specific chelator TPEN completely blocked this pathway (Wang et al., 2015; Jarosz et al., 2017). However, *in vitro*, Zn^2+^ augments monocyte adhesion to endothelial cells, and its deficiency increases the production of proinflammatory cytokines (Jarosz et al., 2017). Therefore, the relationship between Zn^2+^ and inflammation is still under debate, especially in cerebral I/R. The detailed relationship between Zn^2+^ accumulation and inflammation in cerebral I/R needs to be further studied.

To investigate the interaction between Zn^2+^ accumulation and inflammation in the ischemic brain, a rat model of focal cerebral I/R was used in this study. We hypothesize that Zn^2+^ accumulation could exert neurotoxicity effect by promoting inflammation responses in ischemic penumbra of middle cerebral artery occlusion (MCAO) rats. These results provide a novel mechanism for the toxic effect of Zn^2+^ on cerebral I/R injury.

## 2. Materials and methods

### 2.1. Rat model of focal cerebral ischemia/reperfusion

Male Sprague-Dawley (SD) rats (280–300 g) were purchased from SPF Biotechnology Co. (Beijing, China). Animal protocols for these studies were approved by the Institutional Animal Care and Use Committee of Xuanwu Hospital of Capital Medical University. The specific method we used to establish the right MCAO model in SD rats was described previously (Zhao et al., 2014). Briefly, rats were anesthetized with 2% isoflurane in N_2_O:O_2_ (70%:30%). MCAO was induced by the modified intraluminal filament method. The left common carotid artery, internal carotid artery, and external carotid artery branches were exposed by blunt dissection. A microscopic shear is then used to cut an incision in the external carotid artery stump, in which a 4–0 surgical nylon filament with a silicon-coated tip was inserted into the internal carotid artery approximately 18 mm beyond the carotid bifurcation, thereby occluding the origin of the middle cerebral artery. Rats were subjected to 90 min right MCAO followed by 6 or 24 h after reperfusion. Sham-operated rats underwent the same procedure without MCAO. A feedback temperature-controlled heating pad was used during and after surgery to maintain the rat’s body temperature at 37°C ± 0.5°C. Rats were housed in individual cages with free access to food and water (12-h light/dark cycles at 22°C ± 2°C).

### 2.2. Experimental groups, drug administration, and tissue collection

Rats were assigned randomly to three groups: vehicle-treated sham-operated group, vehicle-treated MCAO group, and TPEN-treated MCAO group. Each group was further divided into two subgroups according to different reperfusion time (6 and 24 h; *n* = 6 in each subgroup). TPEN (15 mg/kg dissolved in 10% DMSO) was injected intraperitoneally 30 min before reperfusion. Physiological saline with 10% DMSO was used as control (Zhao et al., 2014).

Brains were harvested and cut into coronal sections with a thickness of 2 mm from 0 to 1.0 bregma. The ipsilateral side of the third slice was processed for Western blotting. The fourth to sixth slices were prepared for 20 μm frozen sections, which were then used for histological staining.

### 2.3. Immunofluorescence and cytosolic labile Zn^2+^ staining

Frozen sections of 20 μm thickness were fixed with 4% paraformaldehyde at room temperature for 10 min. After permeated by 0.5% Triton X-100 and blocked with goat serum, the sections were incubated at 4°C overnight either with the primary antibody against IL-6 (1:200, CST), or with the primary antibody against TNF-α (1:200, CST), NF-κB (1:200, CST), IL-10 (1:200, CST) with mouse monoclonal antibody against NeuN (1:100, CST) respectively. And then, sections were incubated with secondary antibody (1:200, Invitrogen) for 1 h at room temperature. The sections were sealed by dropping the tablet blocking agent containing 4$’$6-diamidino-2-phenylindole (DAPI).

For double staining of cytosolic labile Zn^2+^ with TNF-α, the sections were first incubated with zinc-specific dyes Newport Green (NG; N7991, Invitrogen) for 30 min in the dark as previously described (Zhao et al., 2014). After rinsing in PBS and fixed with 4% paraformaldehyde for 10 min, the sections were incubated with anti-TNF-α antibody (1:200, CST). After incubating with secondary antibody, the nuclei were stained with DAPI and fluorescence was measured with a Nikon fluorescence microscope.

**Figure 1:**
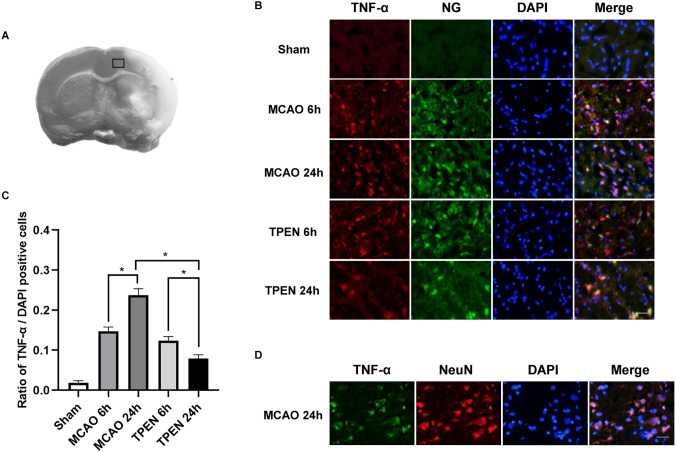
N,N,N’,N’-tetrakis (2-pyridylmethyl) ethylenediamine (TPEN) treatment suppresses the upregulation of inflammatory initiator TNF-α in neurons in ischemic penumbra of middle cerebral artery occlusion (MCAO) rats. **(A)** Brain tissues in black rectangle were observed for histological staining. **(B)** Representative double staining for TNF-α (red) and zinc-specific membrane permeable fluorescent dyes Newport Green (NG; green) in ischemic penumbra at 6 and 24 h after reperfusion. Nuclei were counterstained with 4’,6-diamidino-2-phenylindole (DAPI; blue). **(C)** Quantification of TNF-α-positive cells. Data are expressed as means ± SEMs (*n* = 6). *P < 0.05 vs. the indicated group. **(D)** Representative double staining for TNF-α (green) and neuron-specific nuclear protein (NeuN; red) in the ischemic penumbra of MCAO rats at 24 h after reperfusion. Bars = 20 μm.

For quantitative immunofluorescence, one in every four brain section samples was taken from a continuous series of sections prepared from brain tissue, a total of three sections were taken from each brain. Three areas were selected randomly in black rectangle of Figure 1A from each brain section under 200× magnification and the number of positively stained cells were counted.

### 2.4. Double staining of IL-6 and terminal deoxyribonucleotide transferase dUTP nick end labeling (TUNEL)

The frozen sections were incubated overnight with rabbit polyclonal antibody against IL-6 (1:200, CST). After incubating with secondary antibody (1:200, Invitrogen), a standard TUNEL procedure was performed (in situ Cell Death Detection Kit, POD, Roche Applied Science, Switzerland). The cell nucleus was stained with DAPI and images were acquired using a fluorescence microscope.

### 2.5. Western blotting analysis

Ipsilateral brain tissue slices collected 6 or 24 h after reperfusion were homogenized in RIPA buffer containing protease inhibitors. Protein concentration was determined by a BCA protein assay kit (Pierce, Rockford). Protein samples were separated by 10% SDS-polyacrylamide gel electrophoresis and subsequently transferred to 0.22 μm polyvinylidene difluoride membrane (Millipore). After blocking in 5% skimmed milk for 2 h at room temperature, the membrane was incubated overnight at 4°C with mouse monoclonal antibody against IκB-α (1:1,000; CST). The membrane was then incubated with peroxidase-conjugated goat anti-mouse IgG (1:2,000; Santa) for 1 h and developed with the Super Signal West Pico horseradish peroxidase substrate kit (Pierce). The membrane was re-probed with anti-β-actin antibody (1:4,000; Sigma-Aldrich), which served as a loading control. Expression levels were quantitated by measuring the optical density and expressing the value as a ratio relative to that of β-actin.

### 2.6. Statistical analysis

Data are expressed as mean ± standard errors of the mean (SEMs). Statistical analysis was performed using SPSS version 20.0 (SPSS). The Shapiro–Wilk normality test was used to analyze the normality of data. The data follows a normal distribution were assessed by one-way analysis of variance (ANOVA), followed by *post hoc* least significant difference/Tamhane T2 tests for multiple comparisons. If data did not pass the normality analysis, the data were analyzed using Kruskal-Wallis non-parametric tests. A value of *P* < 0.05 was considered statistically significant.

## 3. Results

### 3.1. TNF-α is colocalized with cytosolic labile Zn^2+^/NeuN following cerebral I/R, and high level of TNF-α is suppressed by TPEN

To investigate the expression of TNF-α, an initiator of inflammatory response, in the ischemic penumbra following cerebral I/R injury and whether chelating Zn^2+^ could inhibit the expression of TNF-α, double immunofluorescence staining was performed by using TNF-α antibody and zinc-specific dye. Figures 1B,C showed that there were very few TNF-α and NG positive cells in the sham group, however, many TNF-α and NG positive cells were observed in the ischemic penumbra of MCAO rats at 6 and 24 h after reperfusion, indicating that cytosolic labile Zn^2+^ accumulation and TNF-α expression occur in the same cells of MCAO rats. Moreover, a drastic reperfusion time-dependent increase of TNF-α-positive cells were observable in the ischemic penumbra at 6 and 24 h after reperfusion, indicating that TNF-α expression keeps on increasing after I/R (*P* < 0.05). TPEN treatment decreased the expression of TNF-α compared to the vehicle-treated MCAO group (*P* < 0.05). Besides, TNF-α-positive cells were displayed NeuN-positive in MCAO rats at 24 h after reperfusion (Figure 1D). These results indicate that ischemia-induced Zn^2+^ accumulation could increase TNF-α expression in neurons, which might aggravate I/R injury.

### 3.2. Chelating Zn^2+^ increases IκB-α expression following I/R injury

**Figure 2:**
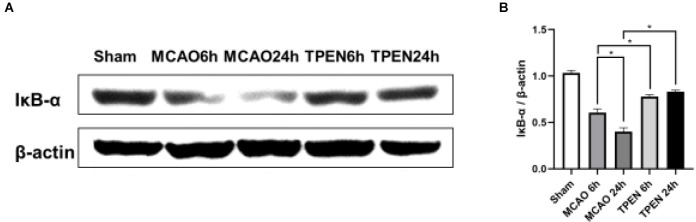
N,N,N’,N’-tetrakis (2-pyridylmethyl) ethylenediamine (TPEN) treatment suppresses the downregulation of inhibitory protein IκB-α of NF-κB in the ischemic hemisphere of middle cerebral artery occlusion (MCAO) rats. **(A,B)** Ipsilateral brain tissue at 6 and 24 h after reperfusion were examined for the expression of IκB-α by Western blotting, with β-actin used as a loading control. Data are expressed as means ± SEMs (*n* = 6). *P < 0.05 vs. the indicated group.

IκB-α is an inhibitor of NF-κB, which binds to and maintains NF-κB in an inactive state in the cytoplasm. Upon stimulation, IκB-α is rapidly downregulated, resulting in the nuclear translocation of NF-κB and initiation of target gene transcription (Traenckner et al., 1994). In order to explore whether Zn^2+^ accumulation influences the level of IκB-α, we measured the expression of IκB-α by Western blotting. The results showed a reperfusion time-dependent decrease of IκB-α expression in MCAO rats at 6 and 24 h after reperfusion (Figures 2A,B, *P* < 0.05). TPEN treatment increased the expression of IκB-α at 6 and 24 h after reperfusion as compared to the vehicle-treated MCAO group (*P* < 0.05), implying that Zn^2+^ accumulation promotes IκB-α degradation after cerebral ischemia.

### 3.3. Nuclear translocation of NF-κB increases in neurons after I/R injury and chelating Zn^2+^ by TPEN reduces the nuclear translocation of NF-κB in ischemic rats

**Figure 3:**
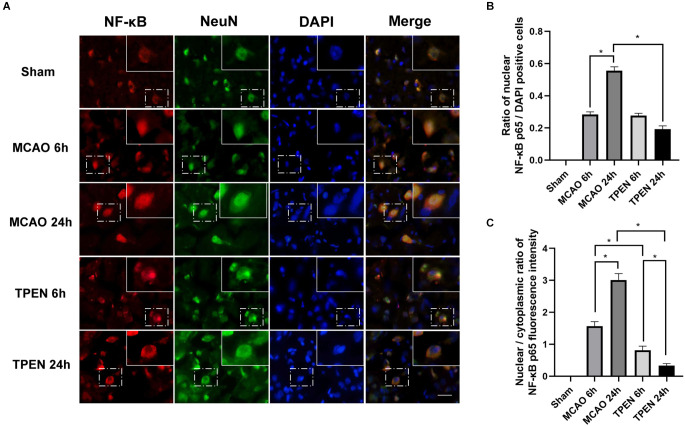
N,N,N’,N’-tetrakis (2-pyridylmethyl) ethylenediamine (TPEN) treatment suppresses the nuclear translocation of NF-κB p65 in neurons in the ischemic penumbra of middle cerebral artery occlusion (MCAO) rats. **(A)** Representative double staining for NF-κB p65 (red) and neuron-specific nuclear protein (NeuN; green) in ischemic penumbra at 6 and 24 h after reperfusion. Nuclei were counterstained with 4’,6-diamidino-2-phenylindole (DAPI; blue). Bar = 20 μm. The images in the dashed-line rectangles are magnified in the solid-line rectangles, showing the nuclear translocation of NF-κB p65 in the ischemic penumbra of MCAO rats. **(B)** The ratio of nuclear NF-κB p65-positive cells was determined. **(C)** The nuclear/cytoplasmic ratio of NF-κB p65 immunofluorescence intensity was determined. Data are expressed as means ± SEMs (*n* = 6). *P < 0.05 vs. the indicated group.

The translocation of NF-κB from cytoplasm to nucleus is known to cause NF-κB activation (Zhang et al., 2005). Figure 3A showed that NF-κB-positive cells were colocalized with NeuN-positive cells in ischemic penumbra of MCAO rats, indicating that ischemia-induced inflammation occurs in neurons. Most of NF-κB p65 was localized in the neuron cytoplasm in the sham group. However, with the increase of reperfusion time, NF-κB p65 increasingly translocated to the neuronal nucleus in the ischemic penumbra of MCAO rats at 6 and 24 h after reperfusion (Figures 3A–C, *P* < 0.05), indicating that inflammatory cytokine NF-κB p65 is activated by I/R injury in ischemic neurons. To investigate the interaction between cytosolic labile Zn^2+^ accumulation and inflammation, Zn^2+^ chelator TPEN was used to remove cytosolic labile Zn^2+^ in MCAO rats. The results showed that TPEN treatment significantly reduced the number of nuclear NF-κB p65-positive cells and the nuclear/cytoplasmic ratio of NF-κB p65 fluorescence intensity compared with the vehicle-treated MCAO group (*P* < 0.05). Taken together, these results indicate that Zn^2+^ accumulation after cerebral ischemia promotes nuclear translocation of NF-κB, which aggravates inflammatory response.

### 3.4. Inflammation-specific apoptosis is occurred in ischemic penumbra, and chelating Zn^2+^ by TPEN suppresses the expression of IL-6 in ischemic rats

**Figure 4:**
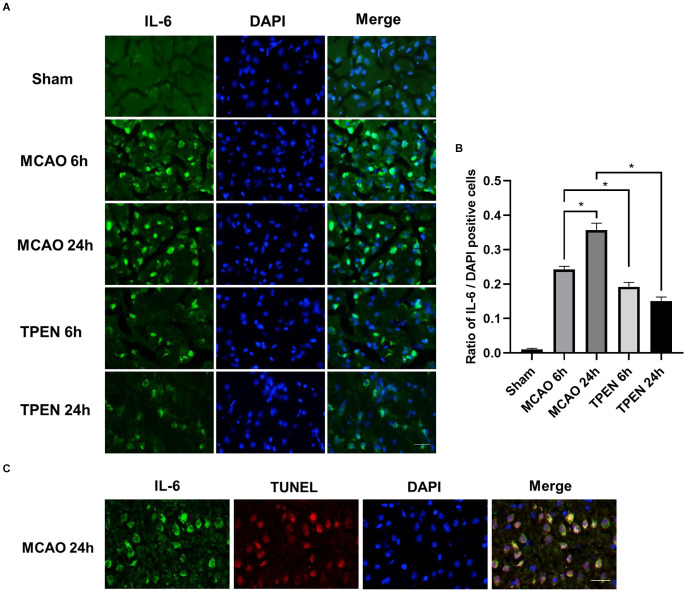
N,N,N’,N’-tetrakis (2-pyridylmethyl) ethylenediamine (TPEN) treatment suppresses the upregulation of proinflammatory cytokine IL-6 in the ischemic penumbra of middle cerebral artery occlusion (MCAO) rats, and IL-6-positive cells are colocalized with terminal deoxyribonucleotide transferase dUTP nick end labeling (TUNEL)-positive cells in the ischemic penumbra of MCAO rats. **(A)** Representative images of brain sections labeled with antibody against IL-6 (green). Nuclei were counterstained with 4’,6-diamidino-2-phenylindole (DAPI; blue). **(B)** Quantification of IL-6-positive cells. Data are expressed as means ± SEMs (*n* = 6). *P < 0.05 vs. the indicated group. **(C)** Representative double staining for IL-6 (green) and TUNEL-stained (red) in the ischemic penumbra of MCAO rats at 24 h after reperfusion. Bars = 20 μm.

To further gain insight into the effects of Zn^2+^ on inflammation, the expression of IL-6 in different groups was assessed by immunofluorescence. There were few IL-6-positive cells in the sham group. However, a drastic reperfusion time dependent increase of IL-6-positive cells was observed in the ischemic penumbra of MCAO rats at 6 and 24 h after reperfusion (Figures 4A,B, *P* < 0.05). TPEN treatment decreased IL-6-positive cells vs. vehicle-treated MCAO group (*P* < 0.05). Besides, IL-6-stained cells were displayed TUNEL-positive in MCAO rats at 24 h after reperfusion (Figure 4C). These results suggest that Zn^2+^ accumulation promotes inflammation responses by upregulating proinflammatory cytokines, leading to inflammation-specific apoptosis in ischemic rats.

### 3.5. Chelating Zn^2+^ increases the expression of anti-inflammatory cytokines in neurons in ischemic penumbra

**Figure 5:**
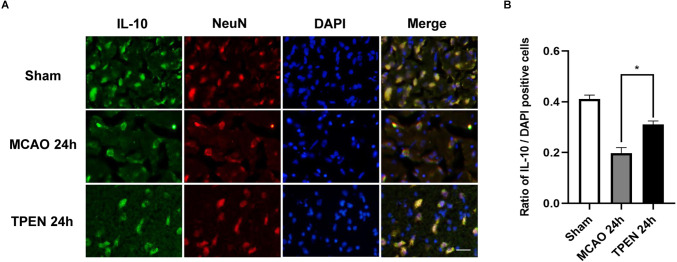
N,N,N’,N’-tetrakis (2-pyridylmethyl) ethylenediamine (TPEN) treatment suppresses the down-regulation of anti-inflammatory cytokine IL-10 in neurons in the ischemic penumbra of middle cerebral artery occlusion (MCAO) rats. **(A)** Representative double staining for IL-10 (green) and neuron-specific nuclear protein (NeuN; red) in ischemic penumbra at 24 h after reperfusion. Nuclei were counterstained with 4’,6-diamidino-2-phenylindole (DAPI; blue). Bar = 20 μm. **(B)** Quantification of IL-10-positive cells. Data are expressed as means ± SEMs (*n* = 6). *P < 0.05 vs. the indicated group.

To investigate the expression of anti-inflammatory cytokine IL-10 in neurons following cerebral I/R injury and whether chelating Zn^2+^ could affect the expression of IL-10, double immunofluorescence staining was performed by using IL-10 and NeuN antibodies. Figures 5A,B showed that IL-10-positive cells were colocalized with NeuN-positive cells, and there were a large number of IL-10 and NeuN-positive cells in the sham group, while a small number of IL-10 and NeuN-positive cells were observed in the ischemic penumbra of vehicle-treated MCAO rats, indicating that ischemia decreased the level of anti-inflammatory cytokine in neurons. TPEN treatment increased the expression of IL-10 after 90 min ischemia and 24 h reperfusion as compared to the vehicle-treated MCAO group (*P* < 0.05). These results suggest that Zn^2+^ accumulation decreases the expression of anti-inflammatory cytokines in ischemic neurons.

## 4. Discussion

In the present study, we investigated whether the Zn^2+^ induces ischemic brain injury by regulating inflammation. We demonstrated that a drastic reperfusion time-dependent increase of proinflammatory cytokines TNF-α, IL-6, and NF-κB p65 nuclear translocation in MCAO rats at 6 and 24 h after reperfusion, while IκB-α, the inhibitory protein of NF-κB, as well as the anti-inflammatory cytokines IL-10 decreased in the ischemic brain tissue of MCAO rats, indicating that cerebral I/R injury triggers inflammation. Importantly, TNF-α-positive cells were colocalized with Zn^2+^ indicator, NG. Treatment with Zn^2+^ chelator TPEN leads to a significant reduction in TNF-α, IL-6, NF-κB p65 expression and an increase in IκB-α and IL-10 expression, indicating that cytosolic labile Zn^2+^ accumulation promotes inflammation following I/R injury. Our results also revealed that TNF-α, NF-κB p65, and IL-10 were colocalized with NeuN-positive cells respectively, and IL-6-positive cells were largely colocalized with TUNEL, indicating that inflammation occurs in neurons of cerebral ischemic rats, which contributes to neuronal death of the brain after cerebral I/R. Taken together, the present study demonstrated that excessive Zn^2+^ accumulation following cerebral ischemia could activate inflammatory mediators and inflammation-mediated neuronal apoptosis, which is a novel mechanism of cerebral ischemic injury.

The inflammatory response was characterized by neutrophil accumulation and proinflammatory factors release (Zhou et al., 2023). It has been reported that neutrophils begin to enter the cerebral cortex through leptomeninges after 6 h of MCAO (Kim et al., 2019), followed by neuronal apoptosis at 24 h after reperfusion (Zhao et al., 2018). Therefore, the expression changes of proinflammatory cytokines and anti-inflammatory cytokine in ischemic rats were studied at 6 and 24 h after reperfusion in this study. TNF-α is a key mediator in neuronal immunomodulatory system, and its abnormal expression plays an important role in various pathological processes (Lambertsen et al., 2012; Li B. et al., 2022). In the present study, very few TNF-α-positive cells were observed in the brain of the sham group, whereas TNF-α-positive cells was gradually upregulated in the ischemic penumbra of MCAO rats at 6 and 24 h after reperfusion, indicating that cerebral I/R resulted in increased expression of TNF-α. Under physiological conditions, Zn^2+^ has been demonstrated to decrease the generation of TNF-α (Prasad et al., 2011; Prasad, 2012; Eddie-Amadi et al., 2022). However, high concentrations of Zn^2+^ in septic shock have been reported to promote the expression of endotoxin-induced TNF-α produced by human monocytes (von Bulow et al., 2007). Our previous study showed that intracellular accumulated Zn^2+^ induced cerebral I/R injury, and chelating Zn^2+^ significantly reduced ischemia-induced cerebral infarct (Zhao et al., 2018). In the present study, we found that TNF-α colocalized with NG and TPEN treatment significantly suppressed the expression of TNF-α in the ischemic penumbra of MCAO rats at 24 h after reperfusion, implying that chelating Zn^2+^ reduces ischemic injury by decreasing the expression of TNF-α in ischemic tissue. Therefore, we speculated that excessive Zn^2+^ accumulation following cerebral I/R might lead to the upregulation of TNF-α, which in turn promote inflammation-mediated brain damage.

NF-κB is a dimer redox-sensitive transcription factor consisting of p50 and p65, and its transcriptional activity is silenced by IκB (Perkins, 2007). Under normal conditions, the NF-κB complex binds to IκB and is sequestered in the cytoplasm. Matsui et al. found that NF-κB is activated after I/R injury in animals (Matsui et al., 2005). As a sequence-specific DNA binding protein, NF-κB was previously reported to be Zn^2+^ dependent (Zabel et al., 1991). Recent studies further reported that Zn^2+^ regulates the activation of NF-κB by acting on TNF-α transcription (Haase et al., 2008). Following stimulation with TNF-α, IκB kinase is activated. The NF-κB dimer becomes active when activated IκB kinase phosphorylates IκB, which leads to ubiquitination and proteasomal degradation of IκB (Chen et al., 1998; Haase et al., 2008). Thus, in the present study, the decreased expression of IκB-α in MCAO rats at 6 and 24 h after reperfusion is possibly due to the increased expression of TNF-α. Chelating Zn^2+^ increased the IκB-α level in MCAO rats, suggesting that Zn^2+^ accumulation-induced increased TNF-α expression might lead to the degradation of IκB-α following cerebral I/R. With the degradation of IκB, NF-κB is released into the nucleus, leading to inflammatory response (Huang and Hung, 2013). In current study, we found that NF-κB p65-positive cells were colocalized with NeuN, NF-κB p65 mostly translocated to the neuronal nucleus in the ischemic penumbra of MCAO rats at 6 and 24 h after reperfusion, suggesting that NF-κB pathway is activated in neurons of ischemic rats. Moreover, nuclear NF-κB p65-positive cells were attenuated by TPEN treatment after reperfusion. Therefore, we speculated that excessive Zn^2+^ accumulation upregulates the expression of TNF-α, which in turn downregulates IκB, leading to the activation of NF-κB to promote neuronal apoptosis following I/R injury.

IL-6 is commonly regarded as a prominent mediator of the inflammatory response, which is involved in the elicitation of acute-phase inflammatory reactions/responses (Kumari et al., 2016; Rasmussen et al., 2019; Ridwan et al., 2021). During inflammatory response, IL-6 expression might be activated by TNF-α (Kumari et al., 2016; Hotter et al., 2019; Jenny et al., 2019; Lambertsen et al., 2019). Besides, it has been reported that Zn^2+^ levels affect IL-6 in DMBA-induced breast cancer in female rats (Gulbahce-Mutlu et al., 2021). In the present study, we found a reperfusion time-dependent increase in IL-6 expression in the ischemic penumbra of MCAO rats, which shows a similar trend with TNF-α. TPEN treatment decreased the expression of IL-6 in ischemic penumbra of MCAO rats at 6 and 24 h after reperfusion, indicating that inflammatory factor levels is associated with the reperfusion time in MCAO rats and the zinc-induced increased expression of TNF-α promotes the expression of IL-6, aggravating the inflammatory response of ischemic tissue. In addition, the results of this study showed that IL-6-positive cells were colocalized with TUNEL at 24 h after reperfusion, suggesting that Zn^2+^ accumulation exacerbates I/R injury through inflammatory response and inflammation-mediated neuronal apoptosis.

IL-10 inhibits the production of proinflammatory cytokines as shown by the development of hyper-inflammation and autoimmunity in IL-10-deficient mice (Frangogiannis et al., 2000; Yao et al., 2008; Jung et al., 2017). In animal models of cerebral ischemia, IL-10 significantly reduced infarct size and neurological deficits (Ooboshi et al., 2005; Jung et al., 2017). Our study found that IL-10-positive cells were largely colocalized with NeuN, and IL-10 expression decreased at 24 h after I/R compared with the sham group, indicating that along with the expression of proinflammatory factors increasing, the expression of anti-inflammatory factors decreased after cerebral ischemia. Besides, we found an obvious increase in IL-10 levels after TPEN treatment, suggesting that inhibition of Zn^2+^ accumulation has anti-inflammatory effect. Therefore, we speculated that high concentrations of Zn^2+^ might influence the expression of anti-inflammatory factors to promote inflammatory response, which in turn aggravates I/R neuronal injury. Although we demonstrated that ischemia-induced Zn^2+^ accumulation in MCAO rats could exacerbate cerebral I/R injury by promoting inflammation, further studies on the cellular and ionic mechanisms underlying the effects of Zn^2+^ on inflammatory factors should be investigated.

In summary, our results clearly demonstrated that cerebral I/R injury induced the upregulation of proinflammatory factors at 6 and 24 h post reperfusion, and downregulated anti-inflammatory factor at 24 h post reperfusion. More importantly, ischemia induced high concentration of Zn^2+^ caused inflammatory apoptosis in neurons, indicating that Zn^2+^ accumulation is an important inducement of inflammatory response and brain injury in ischemic stroke. Our study provided the evidence that Zn^2+^ accumulation following cerebral ischemia induced neuronal inflammation, and inflammation-mediated neuronal apoptosis, which provides an important mechanism of brain injury by Zn^2+^ following cerebral ischemia.

## Data availability statement

The datasets presented in this study can be found in online repositories. The names of the repository/repositories and accession number(s) can be found in the article.

## Ethics statement

The animal study was reviewed and approved by the Institutional Animal Care and Use Committee of Xuanwu Hospital of Capital Medical University.

## Author contributions

WL: investigation, methodology, visualization, writing—original draft. XY: data curation, visualization, methodology, and investigation. MD: data curation, resources, and investigation. WS: investigation, writing—review and editing. YH: investigation, resources. QA: investigation. ZQ: supervision, writing—review and editing. YZ: conceptualization, supervision, resources, methodology, writing—review and editing. All authors contributed to the article and approved the submitted version.

## Funding

This study was supported by grants from the National Natural Science Foundation of China (No. 81971095, 82271308).

## Conflict of Interest

The authors declare that the research was conducted in the absence of any commercial or financial relationships that could be construed as a potential conflict of interest.

## Publisher’s note

All claims expressed in this article are solely those of the authors and do not necessarily represent those of their affiliated organizations, or those of the publisher, the editors and the reviewers. Any product that may be evaluated in this article, or claim that may be made by its manufacturer, is not guaranteed or endorsed by the publisher.
